# Industrial Hemp (*Cannabis sativa* subsp. *sativa*) as an Emerging Source for Value-Added Functional Food Ingredients and Nutraceuticals

**DOI:** 10.3390/molecules25184078

**Published:** 2020-09-07

**Authors:** H. P. Vasantha Rupasinghe, Amy Davis, Shanthanu K. Kumar, Beth Murray, Valtcho D. Zheljazkov

**Affiliations:** 1 Department of Plant, Food, and Environmental Sciences, Faculty of Agriculture, Dalhousie University, Truro, NS B2N 5E3, Canada; A.Unicomb@dal.ca (A.D.); bt673844@dal.ca (B.M.); 2 Section of Horticulture, School of Integrative Plant Science, College of Agriculture and Life Sciences, Cornell University, Ithaca, NY 14850, USA; sk3256@cornell.edu; 3 Department of Crop and Soil Science, 431A Crop Science Building, 3050 SW Campus Way, Oregon State University, Corvallis, OR 97331, USA; Valtcho.jeliazkov@oregonstate.edu

**Keywords:** hemp seed oil, *Cannabis sativa*, health benefits, oil extraction, cannabinoids, CBD

## Abstract

Industrial hemp (*Cannabis sativa* L., Cannabaceae) is an ancient cultivated plant originating from Central Asia and historically has been a multi-use crop valued for its fiber, food, and medicinal uses. Various oriental and Asian cultures kept records of its production and numerous uses. Due to the similarities between industrial hemp (fiber and grain) and the narcotic/medical type of Cannabis, the production of industrial hemp was prohibited in most countries, wiping out centuries of learning and genetic resources. In the past two decades, most countries have legalized industrial hemp production, prompting a significant amount of research on the health benefits of hemp and hemp products. Current research is yet to verify the various health claims of the numerous commercially available hemp products. Hence, this review aims to compile recent advances in the science of industrial hemp, with respect to its use as value-added functional food ingredients/nutraceuticals and health benefits, while also highlighting gaps in our current knowledge and avenues of future research on this high-value multi-use plant for the global food chain.

## 1. Introduction

**Figure 1:**
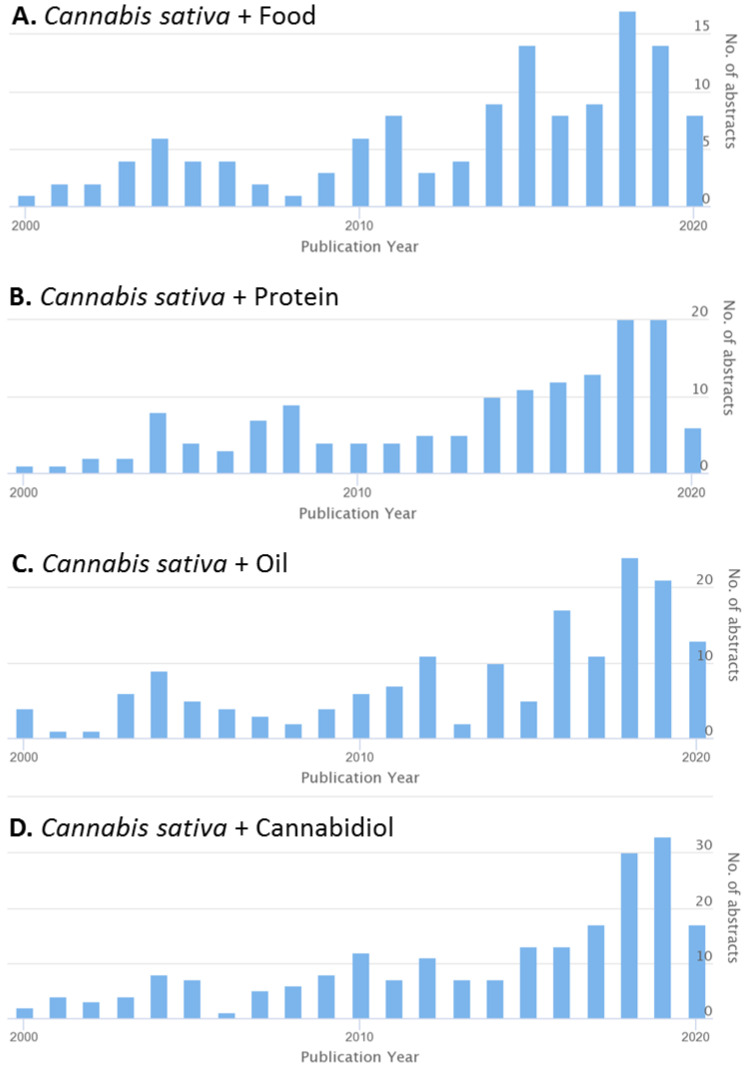
Number of abstracts in the CAB international database in the last 20 years. The search with the keywords (**A**) *Cannabis sativa* + Food, (**B**) *Cannabis sativa* + Protein, (**C**) *Cannabis sativa* + Oil, (**D**) *Cannabis sativa* + Cannabidiol.

Industrial hemp (*Cannabis sativa* L., Cannabaceae) is a versatile herbaceous crop that has been used for fiber, food, and medicinal purposes [1,2]. The cultivation of hemp dates back to China around 2700 BC and is believed to have then expanded across Asia, making its way to Europe 2000–2200 years ago [3,4]. Historically, a multitude of products has been derived from the seeds, fiber, and wooden core of the hemp plant [5]. As a traditional fiber crop, hemp is said to have lined the spine of the first copy of the Bible and set Columbus’s sails with canvas and rope [3,4,5]. As a multi-use crop, hemp is considered one of the oldest plants cultivated to provide nutritional and medicinal benefits [2,6]. The hemp seed, be it raw, cooked, or pressed into oil, has been well documented as a primitive source of fiber, protein, and fat, with high nutritional value [3,6]. Furthermore, properties of hemp have been used to aid in treating and preventing ailments for thousands of years in traditional oriental medicine [3,4]. In recent years, the interest in investigating the potential use of industrial hemp in food and nutraceuticals has been growing (Figure 1).

### 1.1. Botany

Most researchers consider that Cannabis has only one species, *C. sativa* L. In the 1970s, Small and Cronquist [7] separated it into two subspecies: subsp. *indica*, with relatively high amounts of the psychoactive constituent delta-9-tetra-hydrocannabinol (THC), and subsp. *sativa*, with low amounts of THC. The two subspecies can be further broken down into wild and domesticated varieties; under subsp. *sativa*, var. *sativa* is domesticated and var. *spontanea* is wild, and under subsp. *indica*, var. *indica* is domesticated and var. *kafiristanica* is wild [7]. According to these systematics, the modern industrial hemp varieties would belong to subsp. *sativa*, and most medical Cannabis (also called “marijuana”) varieties would belong to subsp. *indica*. However, there are numerous hybrids blurring the line. A contradiction to the above observation has also been reported [8]. Hemp and medical Cannabis strains with 100% *C. indica* ancestry possessed higher genetic variance than strains with 100% *C. sativa* ancestry. Another study using Random Amplified Polymorphic DNA markers of hemp and medical Cannabis also indicated that hemp aligns more with *C. indica* than *C. sativa* [9]. Hillig [10] also strongly opposed the *C. sativa* classification of hemp due to many Asian hemp accessions exhibiting more commonalities with *C. indica*. Apart from indicating a high admixture between the *C. sativa* and *C. indica* genetic pools, these results also suggest that the genetic lineage analysis did not conform to the currently recognized classification, and we may have to revisit the taxonomy of these species to better reflect new genetic information coming to light.

### 1.2. Sex Expression

Hemp is typically a dioecious, obligate cross-pollinated species with a diploid genome (2*n* = 20), although monoecious types have been bred. It is genetically complex and therefore has significant variability in phenotype and sex expression [11,12]. Also, research has shown significant intra- and inter-cultivar karyotype variation among eight monoecious and two dioecious cultivars [13]. Plants may be entirely female, entirely male, or a gradient of intermediate [14].

### 1.3. The Genetic Basis of the Difference between Hemp and Medical Cannabis

**Figure 2:**
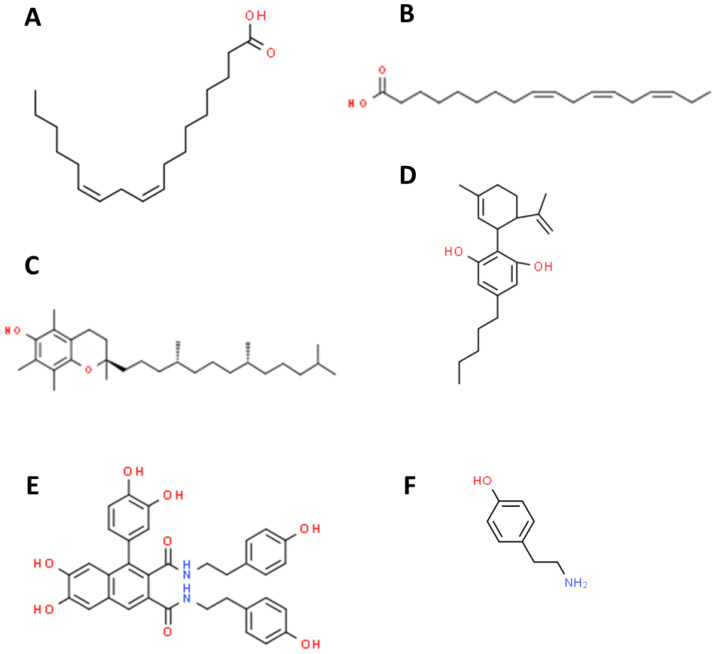
Chemical structures of selected biologically active compounds of industrial hemp. (**A**) Linoleic acid (omega-6 polyunsaturated fatty acid [PUFA]), (**B**) alpha-Linolenic acid (omega-3 PUFA), (**C**) Tocopherol, (**D**) Cannabidiol (CBD), (**E**) Cannabisin A, and (**F**) Caffeoyltyramine.

Industrial hemp and medical Cannabis have primarily been differentiated by their levels of THC production. The cannabinoids (THC and cannabidiol [CBD, Figure 2]) profile and the morphology of the plant are determined by the interaction of genetics and the environment. Genetically, medical Cannabis possesses the B_T_ allele that encodes for tetra-hydrocannabinolic acid synthase, while hemp produces the B_D_ allele encoding for canabidiolic acid (CBDA) synthase [15]. Furthermore, van Bakel et al. [16] studied the transcriptome of female flowers from hemp and medical Cannabis, concluding that there was an up-regulation in the entire THC production pathway in medical Cannabis compared to hemp. This difference translates to producing upwards of 10% THC in many medical Cannabis samples, whereas most hemp samples have a total THC level of 0.3% or less [17]. Some preliminary studies indicated 27% genetic variation between hemp and medical Cannabis samples using Amplified Fragment Length Polymorphisms and genetic variance in certain genomic regions [18,19].

Recent research indicates genome-wide differences that are not confined to the THC biosynthetic pathway [8]. A principal component analysis plot of 81 medical Cannabis and 43 hemp samples obtained from 14,301 single-nucleotide polymorphisms indicated a clear genetic structural difference between hemp and medical Cannabis samples. The hemp samples were more heterogenous than medical Cannabis, indicating the hemp samples came from a wide genome pool, whereas the medical Cannabis samples had a relatively narrow genetic base [8]. Though there are known genetic structural differences, a detailed examination of the genes involved in differentiation, and their corresponding phenotype changes, will provide more input into the genetic basis of the differences between hemp and medical Cannabis. Hemp is resurging in cultivation and production, so care must be taken to conserve the genetic diversity to ensure the long-term survival of the crop.

This review surveys the composition of hemp (both the major nutritive components and the bioactive phytochemicals), as well as their collective health benefits. The aim of this paper is to provide a comprehensive review of hemp seed as a source of value-added or functional food ingredients that is inclusive of its constituents and the role they play in the prevention and treatment of disorders and diseases.

## 2. Hemp Industrial Products

There are various industrial or economic products of hemp. Industrial hemp comprises fiber and oilseed hemp. Fiber hemp is currently considered a niche crop and is grown in temperate regions. Hemp seed (grain) and its derivatives have also gained popularity among consumers and have multiple uses.

It is estimated that the hemp market entails more than 25,000 products, ranging from textiles, clothing, rope, home furnishings, industrial oils, cosmetics, to food and pharmaceuticals [4,20,21]. The durability and high strength properties of the cellulose-rich fiber from the stalk make it a valuable product for rope, paper, construction, and reinforcement materials [1,3,4,22]. Hemp seeds have high nutritional value and pharmacological properties [2,22]. Within the last decade, hemp seed products have expanded to include a range of food and beverages, nutritional supplements, alternative protein sources, and pharmaceuticals [2,20]. In fact, hemp seed’s utility as a functional food ingredient is currently witnessing a revival of old medicinal applications, as its metabolites have shown potent biological activities [1].

### 2.1. Crop Production

The cultivation of industrial hemp is more efficient and less environmentally degrading than that of many other crops [5]. Hemp can be grown under a variety of agro-ecological conditions and has a capacity to grow quickly, especially after the first 4–5 weeks after emergence, making it an excellent candidate for carbon sequestration [4,5,23]. Hemp grows best in sandy loam with good water retention and drainage at temperatures between 16–27 °C, in nutrient balanced soil (especially nitrogen, phosphorus, potassium, magnesium, copper, and others). The planting density depends on the type of crop. Fiber hemp does well in high density to encourage stalk growth, but oilseed and CBD hemp should be planted farther apart to encourage greater branching and flower yields [23]. Densely seeded fiber varieties may reach 5–6 m tall, while some recent grain varieties may only reach 1–1.2 m tall. Many multiple-use or resin cultivars are intermediate in height. Industrial hemp is either harvested for the stalk or seeds, whereas the flowering buds are collected from the narcotic type cultivars [18,23,24,25]. Selection for a specific final product (fiber, seeds, or products from the inflorescences) is reflected in the plant architecture of available varieties and clones [14]. However, architecture also strongly depends on plant density, day length, and nutrients and moisture available in the soil [26].

As a fiber crop, hemp provides a high yield; it produces 250% more fiber than cotton and 600% more fiber than flax, from the same acreage [5,21]. Due to the fast-growing, dense canopy, fiber hemp is a natural weed suppressor and could be grown without herbicides; it also suppresses levels of fungi and nematodes in the soil and can be grown without fungicides or pesticides [5,21,23,24]. Hemp contributes to the maintenance of soil quality by its anchored roots, which prevent soil erosion and nutrient leaching, may extract nutrients from deeper soil layers, and are effective for phytoremediation by absorbing heavy metal contaminants from the soil and storing them within the plant. The continual shedding of leaves through the growing season adds moist organic matter to the soil [1,4,21]. Because of the functions in improving the soil quality, hemp is a prime candidate to be used for crop rotation programs to improve the yield of the main crop [23]. Despite the historical functionality of this multi-purpose crop, global hemp production declined in the 19th century, and still only comprises about 0.5% of the total production of natural fibers [27].

### 2.2. History of Hemp Production

Industrial hemp has been grown as a commodity fiber crop in North America since the mid-18th century until the 1930s. Hemp fell under the umbrella of “marijuana” in the 1930s, and its production was prohibited in Canada under the Narcotics Control Act [3,22,24]. Industrial hemp production acreage and industry rapidly declined in the USA following the Marihuana (SIC) Tax Act of 1937 [28]. However, with the onset of WWII, prohibition was lifted temporarily, when imports of other sources of fiber were unavailable [3]. As an important historical note, hemp was of such necessity to the war effort that the United States Department of Agriculture (USDA) produced an educational video “Hemp for Victory” to encourage farmers to grow hemp [29]. The prohibition was then renewed after the war, and investments in the industry dwindled and were deferred to other crops [3,21,27]. Hemp production generally ceased in North America but continued to a limited extent in Eastern Europe, China, Soviet Union, France, and Spain, where industrial hemp was not prohibited [28]. Hemp production decreased in Europe and the Americas in the late 19th century due to several factors including the replacement of sail ships with steamships, the availability of abaca fiber and rope, and the availability of other less expensive and softer fibers such as cotton. In addition, synthetic fibers such as polyester, nylon, and acrylic were invented in the 1930s and 1940s, and became major fiber competitors after WWII [28]. In 1998, the 60-year hemp production ban was revoked, and under a closely monitored Industrial Hemp Regulation Program, hemp cultivation commenced in Canada [24].

In terms of prohibition, industrial hemp was guilty by its association with medical Cannabis [22]. As mentioned above, both hemp and medical Cannabis belong to the same plant species *Cannabis sativa* L. but are cultivated differently and vary in their phytochemical constituents [20]. In North America and most of Europe, the industrial hemp must not contain more than 0.3% THC in dried herbage [2,17,20,24]. In some countries such as France, this limit was set at 0.2% THC. In the USA, the 2014 Farm Bill permitted “Institutions of higher education” and state agriculture departments to grow hemp under a pilot program if state law permitted it; however, some production aspects were still subject to Drug Enforcement Administration oversight [30]. Before this, all hemp subspecies and varieties were considered Schedule I controlled substances. The 2018 Farm Bill legalized the production of hemp as an agricultural commodity, removed hemp from the list of controlled substances, and listed it as a covered commodity crop under crop insurance [31].

Currently, fiber and grain hemp are minor crops around the world. However, in the last few years, the production of CBD has made hemp one of the most high-value crops. CBD hemp is becoming a major commodity crop in some states in the USA. Moreover, the increased use of Cannabis in the western world as a psychoactive modulatory drug has changed the public perception of hemp.

### 2.3. Industrial Hemp Market

Globally, the industrial hemp market remains in China, where approximately half of the world’s fiber hemp supply is produced [20]. The resurgence of interest in hemp crop can be attributed to the demand for sustainable agricultural practices, along with the recognition of hemp’s superior fiber content and nutritional profile. Primarily in central and western Canada, 340 cultivation licenses were issued to farmers who grew more than 39,000 acres of industrial hemp in 2011 [24,32]. In 2018, there were over 77,000 acres used for hemp production [33]. Since the beginning of state pilot programs to produce industrial hemp in the USA in 2014, the total acreage has increased from 0 to over 90,000, and the number of license holders increased from 292 to 3852 by 2018 [34]. Since the implementation of the 2018 Farm Bill, the acreage has increased even further, to over 146,000 by the end of 2019. Future economic prospects for the crop are unclear; there is competition for land with other crops as well as with medical Cannabis, which can pose an issue due to its ability to crossbreed with hemp, causing issues with the THC content in both crops. There is also global competition; production is increasing rapidly in many places and may exceed demand, driving down profits for hemp [34].

## 3. Hemp Seed Composition

When hemp cultivars are grown primarily for fiber, harvesting is done at the flowering stage, and seeds are not collected. Recently, the production of industrial hemp for the seed has gained interest due to the macronutrients and phytochemicals. Hemp seed is a balanced health product with bioactive components that have the capacity to aid health beyond that of basic nutrition [2,3].

### 3.1. Nutrients

The major constituents of hemp seed include easily digestible protein (20–25%), polyunsaturated fatty acid (PUFA), abundant lipids (25–35%), and carbohydrates (20–30%) high in insoluble fiber (Table 1) [2,3,6,22,35,36,37]. Hemp seed protein is well-suited for human and animal consumption, consisting mainly of high-quality, easily digestible proteins edestin, and albumin, which are abundant with essential amino acids [2,3,6,22]. The rich source of PUFA, linoleic acid (LA; omega-6) and alpha-linolenic acid (ALA; omega-3), is favorable and regarded as balanced for human nutrition at a ratio of 3:1 [2,22,38,39]. LA concentrations range from 64 to 72% of the total fatty-acid composition. This range can be a result of the variation of different hemp cultivars, cultivation techniques, as well as processing and storage conditions. These fatty acids must be acquired from the diet, as they are needed for proper nutrition but cannot be synthesized endogenously [2,22,35,40,41]. Nutritional recommendations indicate that 15–20% of daily caloric intake should come from fats, and approximately one-third of these fats should be essential fatty acids in a 3:1 ratio. It is estimated that this dietary goal can be met with three tablespoons of hemp seed oil [42,43].

### 3.2. Phytocannabinoids and Endocannabinoid System

Hemp flowers and herbage contain valuable phytocannabinoids, which are naturally occurring cannabinoids that are unique to the Cannabis plant [17]. All industrial hemp varieties contain THC, CBD, and other cannabinoids, although the concentrations in some varieties are very low to non-detectable. In northern latitudes, industrial hemp has a particularly high content of CBD and low content of THC [43,47]. CBD content is higher than THC, and CBD can be detected at relatively low levels in hemp seed oil (Table 1). This is because the production and storage of CBD and THC are in the glandular structures of the plant. The wide range of CBD content detected (Table 1) is primarily due to the amount of resin retained by the seed coat during processing, as well as the varying hemp varieties and their associated cultivation conditions [1,25,43,44]. However, the presence of CBD, even in trace amounts, is speculated to provide certain health benefits [1,3,43,44].

The biosynthesis of CBD begins with the polyketide pathway and the plastidal 2-C-methyl-D-erythritol 4-phosphate pathway, which lead to the synthesis of olivetolic acid and geranyl diphosphate, respectively. These precursors undergo condensation to form cannabigerolic acid (CBGA), which is then converted to cannabidiolic acid (CBDA). Decarboxylation of CBDA occurs spontaneously or with the addition of heat to form CBD [1,43,47]. The health benefits of hemp are primarily focused around CBD; however, over 100 cannabinoids are reported to present in *Cannabis* species [48]. These phytocannabinoids can be classified into 11 different classes, namely: (−)-delta-9-trans-tetrahydrocannabinol (Δ9-THC), (−)-delta-8-trans-tetrahydrocannabinol (Δ8-THC), cannabigerol (CBG), cannabichromene (CBC), CBD, cannabinodiol (CBND), cannabielsoin (CBE), cannabicyclol (CBL), cannabinol (CBN), cannabitriol (CBT) and miscellaneous-type cannabinoids. Recently, besides THC and CBD, 30 other cannabinoids from commercial hemp seed oil have been identified using high-resolution mass spectrometry [49].

The endocannabinoid system of humans is an endogenous signaling system consists of endocannabinoids, enzymes involved in their synthesis and degradation, cannabinoid receptors, and other associated elements [50,51]. The system is modulated by diet, sleep, exercise, stress, among many others. The endocannabinoids are fatty-acid-derived neurotransmitters that act as signal molecules of coordinating intercellular communication across all physiological systems. One of the primary functions of the system is to restore homeostasis following cellular stressors. The two most studied endocannabinoids are anandamide-*N*-arachidonylethanolamine (AEA) and 2-arachidonylglycerol (2-AG). Phytocannabinoids are recognized as pharmacologically active compounds, which function by interacting with the endocannabinoid system in humans [1,52]. Cannabinoid receptors are 7-transmembrane-domain G-protein-coupled receptors. Two cannabinoid receptors have been identified: the central CB1 receptor and the peripheral CB2 receptor [53]. The CB1 receptor is primarily present in the brain and spinal cord but also found on certain cells of the immune system, adipose tissues, liver, muscle, reproductive cells, kidney, and lungs. CB1 mediates the release of neurotransmitters such as acetylcholine, noradrenaline, dopamine, gamma-aminobutyric acid (GABA), and glutamate. The CB2 receptor is expressed mainly in the cells in the periphery, in the organs of the immune system and have a role in the release of cytokines and the modulation of immune cell migration [53,54,55] but not psychoactivity [54]. The diversified physiological effects of endocannabinoids take place when they bind to and activate these receptors.

The pharmacology or interaction of THC and CBD with the endocannabinoid system is not yet fully understood and seems complicated. THC has been shown to provide most of the psychoactive effects through the CB1 receptor as an agonist; however, under certain conditions, THC act as an antagonist of the CB1 receptor and also shown to interact with CB2 receptor [54,55]. Interaction of THC with CB1 receptor inhibits ongoing neurotransmitter release; however, repeated administration of THC may nullify its effect as well as the action of endocannabinoids [55]. In contrast, CBD has minimal direct activity at CB1 and CB2 receptors; therefore, no psychoactive activity similar to THC. Though CBD has a very low affinity for CB1 and CB2 receptors, CBD can bind these receptors [56]. CBD antagonizes synthetic agonists of CB1 and CB2 receptors and can be considered to be a negative allosteric modulator of CB1 and CB2 receptors. Moreover, recent findings also indicate that CBD exhibits various dose-dependent physiological responses. Though the low doses (30 mg oral) has no intoxicating effects, high doses (300 mg oral) increased somnolence and reduced anxiety [55]. Moreover, the biological activity of CBD seems to be complex due to its complex pharmacological actions, such as inhibition of endocannabinoid reuptake and increasing the activity of serotonin 5-HT_1A_ receptors, binding to non-cannabinoid receptors such as transient receptor potential vanilloid 1 (TRPV1), peroxisome proliferator-activated receptor-γ (PPARγ), and the orphan receptor G protein-coupled receptor 55 (GPR55) [51,55,57]. CBD has recently received increasing interest since chronic administration of CBD has shown potential therapeutic properties such as antiepileptic, anxiolytic, antipsychotic, neuroprotective activities, and benefits against disorders of motility and epilepsy [55,56,58].

### 3.3. Hemp Seed Oil

Hemp seed oil contains tocopherol isomers beta-tocopherol, gamma-tocopherol, alpha-tocopherol, and delta-tocopherol, with the gamma-tocopherol derivative present in the highest quantity (Table 1) [2,41,45]. Tocopherols are natural antioxidants that can reduce the risk of oxidative degeneration related disorders [2,41]. In addition, terpenes and polyphenols have been detected, which contribute to the odor/flavor and intrinsic antioxidant activity, respectively [1,2]. Among phenolic compounds, flavonoids, such as flavanones, flavonols, flavanols, and isoflavones were the most abundant [46]. The reported phytochemical contents of hemp seed oil vary due to a broad range of existing hemp cultivars, which are grown and processed under diverse conditions.

## 4. Potential Health Benefits

Numerous health benefits and potential therapies are reported for hemp seed. Hemp seed delivers a desirable ratio of omega-6 to omega-3 PUFA (Figure 2), which can improve cardiovascular health, reduce osteoporosis symptoms, and diminish eczema conditions. CBD exerts pharmacological properties that make it a potential therapeutic agent for central nervous system diseases, such as epilepsy, neurodegenerative diseases, and multiple sclerosis (MS) [1,59].

### 4.1. Cardiovascular Health 

The dietary intervention of hemp seed for cardiovascular health has been examined. Schwab et al. [60] supplemented the human diet with 30 mL of hemp seed oil daily for four weeks and detected positive changes in the serum lipid profile. Another study also noted that rats fed a 5% or 10% hemp seed-supplemented diet for 12 weeks experienced an elevation in plasma LA and ALA levels [61]. After the diet, post-ischemic heart performance was assessed; the heart’s ability to recover from ischemia-reperfusion insult appeared to be directly related to the hemp seed’s PUFA. Richard, Ganguly, Steigerwald, Al-Khalifa, and Pierce [62] also found that the integration of hemp seed into the rat diet significantly increased plasma LA and ALA levels. As a result, platelet aggregation was inhibited and slowed to a lower rate. The diminished likelihood of clot formation has implications for reducing the incidences of myocardial infarctions and strokes [62]. Prociuk et al. [63] reported similar findings after examining the effect of dietary hemp seed for eight weeks in rabbits. Elevated plasma levels of PUFAs indirectly decreased the risk of platelet aggregation and myocardial infarction and provided better defense against hypercholesterolemia [63]. Other issues caused by hypercholesteremia that were improved by supplementing hemp seed, including decreased cholesterol, low-density lipoprotein, and triglyceride levels, increased high-density lipoprotein levels, lower plaque, and fat deposition, and lower arterial wall damage [64].

### 4.2. Cancers

Since the first study exhibiting the anti-cancer effects of Cannabis phytochemicals by Munson, Harris, Friedman, Dewey, and Carchman [65], there have been major advances in understanding the mechanisms and targeting action of cannabinoids. Evidence suggests that phyto-, endo-, and synthetic cannabinoids contain properties that aid in the treatment of the brain, prostate, breast, skin, pancreas, and colon cancer. Both in vitro and in vivo models suggest cannabinoids play a role in regulating cellular mechanisms causing anti-proliferative, anti-metastatic, anti-angiogenic, and pro-apoptotic responses [66,67]. These findings have major implications in oncology, as it has been well established that most cancers originate from uncontrolled or improperly managed cellular growth [67].

Phytocannabinoids demonstrate the potential to inhibit cell growth and induce apoptosis in gliomas. Massi et al. [53] tested the effect of introducing CBD to U87 and U373 human glioma cell lines. In vitro treatment resulted in a reduction in mitochondrial oxidative metabolism and glioma cell viability. It was also confirmed that CBD induced apoptosis. When a CB2 receptor antagonist was introduced to the glioma cell lines, the antiproliferative effect of CBD was hindered, revealing its mechanism of action [53]. Vaccani, Massi, Colombo, Rubino, and Parolaro [68] also looked at the implications of CBD on the U87 glioma cell line, where an anti-metastatic result was observed due to the inhibition of cell migration. Cannabinoids have also been found to prevent the differentiation and proliferation of glioma stem-like cells, which may help treat the difficult-to-eliminate nature of gliomas [69].

The treatment of prostate and breast cancers with CBD have also been explored. Sarfaraz et al. [67] found that androgen-responsive human prostate carcinoma cells treated with CBD exhibited a pro-apoptotic response, inhibited cell growth, and a lowered secretion of prostate-specific antigen, which is typically elevated in cancerous cells [67]. Of several natural cannabinoids tested, a CBD extract provided the most potent cytotoxic effects against breast cancer cells, with significantly lower damage to healthy cells [70]. CBD induced apoptosis in a breast cancer cell line via the activation of the overexpressed CB2 receptor [70,71].

Other studies have explored cannabinoid therapy in skin, pancreas, and colon cancers. Blázquez et al. [72] evaluated cannabinoid receptor agonists in mice and found that the activation of these receptors decreased the growth, proliferation, angiogenesis, and metastasis of melanomas. Through similar actions, cannabinoids induced apoptosis in pancreatic tumor cell lines, and the effects were lessened when the CB2 receptors were blocked [73]. Promising results were found in vivo by Ferro et al. [74], where mice with pancreatic ductal adenocarcinoma treated with gemcitabine and CBD survived nearly three times as long as mice treated only with gemcitabine or with a vehicle. This was achieved through interference with the G-coupled protein receptor GPR55, resulting in the prevention of growth and cell cycle arrest [74]. Cianchi et al. [75] investigated the activation of the cannabinoid receptors in colorectal cancer and demonstrated similar apoptotic mechanisms to pancreatic and melanoma cancers.

The strategic elimination of these cancer cells, while inflicting limited harm to normal cells, shows potential for CBD mediation. Although the range of cancers therapeutically affected by cannabinoids is promising, further investigations are required to interpret the growth-inhibitory action of CBD. The results presented here reinforce that much of the CBD effect is mediated through the activation of CB2 receptors and that the possible application of CBD in cancer cytotoxicity is vast.

### 4.3. Diseases of the Central Nervous System

Several phytocannabinoids have exhibited the ability to mediate symptoms of neurodegenerative diseases and reduce compromising damage. Hypoxic-ischemic (HI) brain injury results when the brain is deprived of oxygen and can lead to neurological impairments such as epilepsy, developmental delay, as well as reduced motor and cognitive function. Castillo, Tolón, Fernández-Ruiz, Romero, and Martinez-Orgado [76] found that CBD enhanced neuroprotection in mice that experienced induced HI by oxygen and glucose withdrawal. Pazos et al. [77] tested rats that underwent HI injury and subsequently received CBD treatment; the common measures of HI damage, infarct volume, and histological evaluation indicated CBD provided neuroprotection. Later, Pazos et al. [78] studied HI in a pig model by reducing carotid blood flow and then administering CBD treatment [78]. The neuroprotective action was attributed to the prevention of an increase in excitotoxicity, oxidative stress, and inflammation, and CB2 receptors were associated with these effects [77,78]. Treatment with CBD prevents emotional and cognitive impairments, injury to white matter, degeneration of hippocampus tissue, and glial cell response decrease that result from brain ischemia, as well as promotes recovery through hippocampus dendritic cell reconstruction and neurogenesis in mice that already have brain ischemia [79].

The most prevalent neurological disease, epilepsy, has also benefited from CBD. Jones et al. [80] examined seizure activity and found that CBD exerted anticonvulsant properties. Jones et al. [81] reconfirmed these findings using an acute pilocarpine model of temporal lobe seizure and the penicillin model of the partial seizure. Both studies found a decrease in both the severity and mortality of the seizures [80,81]. Intervention with CBD is even beneficial to people who have treatment-resistant epilepsy; adverse events, severity, and frequency of seizures were significantly and sustainably reduced with long-term treatment [82].

Several clinical studies have outlined the cannabinoid treatment of spasticity, pain, and hindered bladder control symptoms associated with MS patients. A novel cannabinoid therapy, THC/CBD oromucosal spray (Sativex™), has been introduced to patients suffering from neuropathic pain that can be difficult to manage with normal pharmaceuticals. A placebo-controlled study found that the spray was able to lessen MS-induced neuropathic pain [83]. The same spray was evaluated for symptomatic relief and was found to cause a decline in spasticity occurrence and severity, and had limited adverse side effects on cognition [84]. This could be due to the critical part CBD plays in diminishing the psychoactive effects of THC. A similar spray provided to MS patients effectively reduced pain and sleep disturbance [85]. When MS patients were provided with THC/CBD extract capsules, daily self-reports of spasm frequency, mobility, and ability to fall asleep were favorably impacted in the active treatment group [86].

Limited research has been done on CBDs effect on Parkinson’s disease symptoms, though the current evidence suggests it can improve the non-mobility related symptoms, there is contradicting evidence on its effects on mobility and cognition symptoms [87]. Further studies need to be conducted to determine the true extent of CBD treatment on Parkinson’s disease.

The mechanisms by which CBD exerts its neuroprotective effects are not entirely understood; however, CBD is noted for its antioxidant and anti-inflammatory properties [76,80]. Since the activation of CB1 receptors is consequently associated with psychoactive ramifications and potentially neurodegenerative symptoms upon long-term activation, the investigation of CBD is increasingly important for neurological disorders. At present, CBD used therapeutically, either alone or in combination with THC, aids in the treatment and symptomatic relief of several neurodegenerative disorders.

### 4.4. Rheumatoid Arthritis

In traditional Chinese folk medicine, hemp seed oil has been used to relieve chronic knee pain in patients with rheumatoid arthritis (RA) and improve blood circulation [88]. RA is an autoimmune inflammatory disease primarily characterized by synovial tissue inflammation and hyperplasia [89]. Jeong et al. [90] concluded that hemp seed oil promotes the production of reactive oxygen species (ROS), storage of lipids, production of endoplasmic reticulum stress markers, which act as anti-rheumatoid factors in downstream processes, and improved blood circulation, providing additional relief to RA patients. Hammell et al. [91] found that CBD can positively impact pain caused by arthritis. A rat model was used to examine topical application of CBD: joint swelling, pain scores, synovial membrane thickness, infiltration of immune cells, and inflammation biomarkers were all significantly reduced in a dose-dependent manner [91]. A CBD-based oil was used to treat another kind of arthritis: osteoarthritis in dogs [92]. Dogs receiving treatment exhibited significantly less pain compared to those without treatment, allowing these dogs to be more comfortable and active [92]. Clinical studies on RA patients will provide clarity on the mechanism and biochemistry behind the benefits of hemp seed oil in reducing and ameliorating the symptoms of RA.

### 4.5. Dermatitis and Skin Diseases

Hemp seed oil can be an effective cure to eczema, as well as a host of other skin related ailments [93]. Hemp seed oil is composed of more than 80% PUFA, and is rich in tocopherols [3,41]. These constituents point to hemp seed oil’s beneficial effects in reducing and eradicating skin diseases, including eczema [94]. A clinical study by Callaway et al. [93] found participants who had a regular dietary intake of hemp seed oil had significantly fewer symptoms of eczema, including skin dryness and itchiness, and they used dermatitis medicine less often. Allergic contact dermatitis has shown preliminary evidence to be mediated through intervention with the endocannabinoid system, making treatment with CBD a promising solution [95]. The presence of high levels of essential PUFAs improves the atopic symptoms of dermatitis [93,96].

### 4.6. Mental Health and Sleep Disorders

Concentrated CBD from hemp has been shown in both pre-clinical and clinical studies to possess anxiolytic or antianxiety characteristics due to its ameliorating effect on limbic and paralimbic areas of the brain [97,98]. Importantly, the anxiolytic effects of CBD are only induced with low concentrations; high concentrations may cause anxiogenic or panicogenic effects [99]. Treatment doses need to be selected carefully to ensure only anxiolytic benefits are felt by the individual. Other anxiety-related disorders also benefit from treatment with CBD, including post-traumatic stress disorder (PTSD) and depression, as well as addiction recovery [99,100,101]. The endocannabinoid system is involved in learning, emotional responses (including those related to trauma), and regulation of emotional behavior; therefore, this system is an important target for the treatment of PTSD [100]. Using experimental animal models, CBD has been effectively used to treat the development of adverse associations at all steps of the process, including immediately after trauma to prevent the development of PTSD. CBD has been able to help in the extinction process of adverse memories in humans, as well as treat the anxiety-related symptoms accompanying PTSD without causing side effects [100]. In male and female genetically depressive mice, CBD had anti-depressant properties as well as reduced the exhibition of anhedonia [102]. In patients at high risk of psychosis, CBD was able to partially normalize function in regions of the brain associated with psychosis [103]. When administered to sober heroin-addicted individuals, CBD reduced cue-induced cravings and anxiety with short-term 3-day administration, as well as had prolonged benefits up to 1 week after the final treatment dose [104].

CBD has been shown to have therapeutic effects in favorably modifying REM sleep behaviors that may be altered due to insomnia [105]. A study conducted on people experiencing anxiety and sleep issues found that CBD improved sleep quality in the first month, but it did not remain constant throughout the remainder of the study period [106]. There are contradictions in the literature, where some studies have found, as discussed, that CBD can improve sleep; however, there are other studies that find treatment with CBD can improve wakefulness during the day [107]. The mechanisms behind sleep cycle regulation by CBD need to be more thoroughly explored to determine how it can be used to improve both sleep and wakefulness.

Comprehensive research on this topic is required to understand the broad-spectrum effects of hemp-seed-derived CBD-based nutraceuticals on anxiety [108]. Data is especially lacking on the differences between sexes in response to treatment; most pre-clinical studies used only male animals, and clinical studies that include females have yet to evaluate sex-differentiated responses [98]. Males and females experience anxiety differently, and they respond to psychotropics differently, so this is an important knowledge gap to fill with further studies [98]. There is also limited research on CBD treatment for the other anxiety-related disorders discussed above. There are contradictions within the literature on the true benefit of CBD on the treatment of addictions, some of the conflicts are due to the type of drug at the center of the addiction, but there is also lacking consensus within drug types [109].

### 4.7. Additional Health Benefits

There are other areas of treatment using hemp products that have been explored less extensively than those discussed above. The hemp seed oil has been documented to be therapeutic for constipation problems [110]. Furthermore, mice trials have shown that hemp seed consumption leads to improved memory and learning-induced by chemical drugs [111,112].

CBD has suppressive effects on the immune system, including inflammatory response reduction, cellular and humoral immunity suppression, and induction of apoptosis in some lymphocytes; these effects are beneficial for treating inflammatory diseases [113,114]. Type 1 diabetes is an example of an inflammatory-based disease that can benefit from CBD preemptive treatment; non-obese diabetic mice receiving CBD had delayed development of diabetes, and had significantly lower activation of leukocytes than mice receiving control vehicle [115]. Zhou, Wang, Ji, Lou, and Fan [116] demonstrated anti-neuroinflammatory properties of hemp seed using an experimental mouse model.

Another area of research on the benefits of hemp is pain management. It has been theorized that some pain conditions, including fibromyalgia, migraine, and irritable bowel syndrome, are caused by an endocannabinoid deficiency [117]. Due to this theory, targeting the endocannabinoid system with CBD is a common treatment for symptomatic relief of these conditions [117]. Cannabis has also commonly been used to treat other chronic pain that is not suspected to be caused by an endocannabinoid deficiency; it is the most common reason for medicinal Cannabis usage in the USA [118,119]. Cannabinoids act in many ways to produce an analgesic effect, including preventing the release of neurotransmitters from presynaptic neurons, altering the sensitivity of postsynaptic neurons, activating pain inhibiting pathways, and reducing neural inflammation [119].

The major limitation for the treatment of all previously discussed health conditions is the lack of long-term studies. There has virtually been no research examining the long-term effects, especially of hemp-derived CBD-based treatments. Short-term data shows that it has been well-tolerated and results in minimal adverse side effects [119]. The cannabinoids and terpenes in Cannabis work synergistically together to provide the discussed health benefits in addition to the flavonoids present [118]. In the future, investigations should be conducted to understand the synergistic effect of all the phytochemicals in addition to validating the health benefits of minor constituents of hemp seed.

## 5. Food and Nutraceutical Applications

Consumers have become increasingly interested in the way their diet can address health deficits and wellbeing. Over a decade ago, two thirds of grocery shoppers reported that their purchases were highly influenced by the pursuit of preventing, managing, or treating a specific health condition [120]. Since then, food scientists have targeted such consumer demands by investigating and advertising additional health benefits and bioactive properties that functional foods provide. In recent years, some unconventional plant-derived oils, such as hemp seed oil, have earned a reputation for providing not only cooking and alimentary services but also providing medicinal and nutraceutical potential [121]. Hemp seed oil is currently advertised primarily as a natural health product for body care purposes, as oil for salad dressings, or to be taken directly as a dietary supplement. The hemp seed oil has a strong susceptibility to rancidity with heat and prolonged storage, which reduces its use as cooking oil [40,120,121]. Because hemp prohibition was only lifted about 20 years ago, only recently that hemp seed has been investigated for its applications in the food and nutraceutical industry for its benefits beyond basic nutrition.

### 5.1. Hemp Seed in Food Products

In addition to the primary use of hemp seed as oil, it has been used in the milled form as a source of vegetable protein and dietary fiber, facilitating its incorporation into food products such as energy bars, flavored yogurt, baked goods, and more [36,122,123]. Shim [124,125] patented a process of making bread and confectionary from hemp seed oil and hemp seeds, respectively. Guang and Wenwei [126] patented hemp seed flours to be used in functional foods that aid in the prevention of certain diseases by increasing the levels of high-density lipoprotein (HDL) and stabilizing the levels of other glycerides and lipoproteins. A seasoning sauce from fermented hemp seeds was developed by Metz and Selg-Mann [127], while Steinbach [128] developed a process for producing pralines and chocolates from hemp seed and hemp seed oil. A process was developed for obtaining hemp milk that did not change color or develop bitterness when subjected to pasteurization [129]. Hemp seed as a powder and an additive has been used as a source of protein [130,131]. Furthermore, Guang and Wenwei [132] developed a process for using hemp protein powder in treating anemia. Though the most popular part of the hemp plant to ingest is the seeds, sprouts, leaves, and flowers can also be consumed raw in juice or salads [133]. The inclusion of juice obtained from hemp in alcoholic beverages is speculated to have digestive benefits [134].

Frassinetti et al. [135] examined hemp seeds and sprouts to be rich in beneficial bioactive compounds with both in vitro and ex vivo antioxidant activities. Furthermore, these compounds exhibited an antimutagenic effect on *Saccharomyces cerevisiae*. The main polyphenols identified in seeds and sprouts exhibiting antioxidant activities were cannabisin A, B, C, and caffeoyltyramine (Figure 2). The two primary compounds identified in sprouts that provide nutraceutical benefits were linoleic acid and gluconic acids, which act as intermediaries in the production of vitamin C [135]. Terpenes, which are also found in hemp, have anti-inflammatory and some antiallergic properties, can treat pain, prevent the production of ROS, and act as potent antioxidants [133]. Due to the presence of a wide variety of nutrients, including high levels of PUFA and essential amino acids, hemp seeds are praised for providing adequate quantities of different nutrients to satisfy human dietary requirements [136,137].

### 5.2. Advancement in the Extraction of Oil and Cannabinoids from Hemp Seed

There are numerous methods for extraction of hemp seed oil, including cold press, supercritical CO_2_ extraction, solvent extraction with isopropanol, hexane, dimethyl ether, and numerous pretreatments. However, all of these methods possess different advantages and disadvantages depending on the end use of the product and the extraction fraction in question [138].

Cold-pressed oils from seeds have become more commercially popular since they are viewed as natural and safe products to be used in food [120,139]. Cold-pressing passes the raw seed material through a conventional screw press, without the addition of harsh chemical solvents or high heat treatments [40,120]. This process retains more of the beneficial components of the seeds, including valuable PUFA and bioactive substances, while minimizing degradative changes in the oil [40,120,121,139]. One notable disadvantage of cold-pressed oil is the low yield potential of 60–80% of extractable oil [6].

Soxhlet extraction is the conventional method of extraction; the selected solvent is heated to reflux and floods the solid material, extracting the desired compounds, including volatile compounds [140]. Many solvents have been successfully used to extract hemp seed oil with high yields. N-hexane and petroleum ether [141], dimethyl ether [142], ethanol [143] and isopropanol [144] have been used and optimized with regards to extraction time, temperature, and other extraction conditions.

Another method optimized recently is supercritical fluid extraction, most commonly using CO_2_. Using the response surface method, Da Porto, Decorti, and Tubaro [145] and Da Porto, Voinovich, Decorti, and Natolino [146] optimized supercritical CO_2_ extraction of hemp oil; they observed fatty-acid compositions and oxidative stability at different stages of the extraction process while varying the parameters to obtain maximum efficiency of extraction. In addition, Aladić et al. [147] and Tomita et al. [148] further refined the processing temperature, pressure, and time to determine how these conditions affect the constituency of hemp oil, especially focusing on fatty acids, tocopherol, and pigment content. Supercritical CO_2_ using n-propane as a solvent, reduces the extraction pressure and preserves the physical and nutritional properties of hemp seed oil [144].

There have been many innovations in hemp seed oil extraction. Optimized procedures to extract hemp seed oil rich in CBD by supercritical CO_2_ are well established [149,150]. To remove pigments and waxes prior to supercritical CO_2_ extraction, crushed silicon sand and ultrasonic-assisted extraction, respectively, can be used [151,152]. Procedures to extract hemp seed oil free of THC have also been developed to satisfy regulatory requirements and societal concerns. Separation techniques such as chromatographic columns, and stabilization reactions such as oxidation with heat and isomerization with UV light, have been reported [153,154]. Dynamic maceration with ethanol for 45 min is an efficient method to extract non-THC cannabinoids from hemp seed oil [155]. New methods using ultrasonication-assisted extraction are also gaining interest due to minimal intervention with the product and shorter extraction time [156,157]. Similarly, the response surface method has been used to optimize the microwave-assisted extraction of cannabinoids, which also provides a shorter extraction time [158]. Recently, many advances have been made to combine different techniques, such as supercritical fluid extraction, ultrasonication, and microwave-assisted extraction, to increase efficiency [156]. Hemp seed oil extracted through the above methods are different in yield, physical properties, and chemical composition. Furthermore, the cost is also an important factor in the selection of the extraction method. Considering an initial economic cost-benefit analysis, supercritical CO_2_ extraction is most efficient, followed by Soxhlet extraction and ultrasonication [138]. In terms of scale-up extraction, ultrasonication and Soxhlet extraction are the best methods, while the desirable omega-6 PUFA/omega-3 PUFA ratio can be achieved by the Soxhlet extraction method [138]. Selecting the most appropriate method of extraction depends on the end use and desired bioactives in the final products.

### 5.3. Methods of Enhancing Oxidative Stability of Hemp Seed Oil

To maintain oxidative stability, it is necessary to monitor the fatty-acid profiles throughout the extraction process to standardize temperature, pressure, and particle size required for supercritical CO_2_ extraction of hemp seed oil [145,146]. Hemp seed oil can maintain oxidative stability through the presence of tocopherols and polyphenols. Tocopherols effectively stop or slow down the lipoperoxidative radical chain reactions by preventing the oxidation of PUFAs [159]. Furthermore, phytosterol concentrations of approximately 15% also have excellent oxidative prevention functions [160]. Among them, b-sitosterol, campesterol, and D_5_–avenasterol can withstand high temperatures and reinforce the plasma membranes of eukaryotic cells. Storage studies must be conducted for hemp seed oil while observing the changes in composition and antioxidant activity.

Some research has been done in improving oxidative stability and adhesion of hemp seed oil to surfaces such as skin or hair. A method of saponizing and quarternizing fatty acids [161] resulted in the minimization of oxidation and crosslinking of released essential fatty acids. Many cosmetic formulations of hemp seed oil were prepared with this method to improve adherence to skin. Maintenance of the antioxidant properties of the oil helps regulate oxidative stability as well. Temperature and pressure play a major role in altering oxidative stability; however, there is no universal standard that specifies the optimal conditions for maintaining oxidative stability as it varies greatly between extraction procedures. Hence, it is more likely that optimization at the local process level will help maintain the oxidative stability of hemp seed oil.

### 5.4. Microencapsulation Technologies

To increase the bioavailability and protect unstable food constituents, such as PUFAs, from oxidation, different types of microencapsulation techniques have been used for plant-based oils [162]. Spray drying [163], freeze-drying [164], fluidized bed coating [165], centrifugal extrusion [166], complex coacervation [167,168], ionotropic gelation [169], liposome entrapment [170], and electrospraying [171] are the most predominant methods used for microencapsulation. Hemp seed oil is a prime candidate for these interventions to increase its nutritional value and benefits. The selection of the shell coating material to protect the core substance during microencapsulation depends on the microencapsulation method, the nature of the core material, the end use of the product, its physicochemical characteristics, and possible interactions with the core material [172].

Nanoencapsulation is remarkable in improving the low water solubility, bioavailability, volatility, and stability of high-value oils [173]. Belščak-Cvitanović et al. [174] concentrated and encapsulated the bioactive compounds extracted from hemp fiber processing waste, also called hemp fiber meal. Hemp fiber meal can be used for isolation of essential amino acids, especially arginine, by using food grade enzymes for polysaccharide digestion; the resulting polysaccharide fragments can be subjected to ultrafiltration and removed to concentrate the protein content, making it a superior isolate compared to other hemp protein products [175].

Considerable evidence of the potential health benefits of hemp seed oil has been uncovered in the past two decades; however, additional investigations are required to use hemp seed oil as a functional food ingredient. The value-added hemp food sector is growing; with increased consumer awareness and product innovation, the health applications of hemp seed oil are expected to expand [24,42].

## 6. Future Prospects and Conclusions

**Figure 3:**
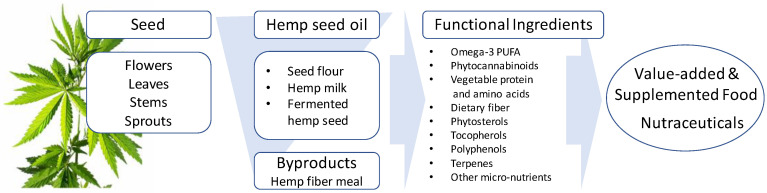
Advanced value-added technologies can drive value-added innovation to make use of industrial hemp to introduce a wide array of functional food ingredients and nutraceuticals.

Since ancient times, hemp has been cultivated to provide nutritional and medicinal benefits. Although the government regulations repressed the cultivation and scientific inquiry of industrial hemp in the past, under recent legalization with stringent production regulations, hemp has proven to hold viable, value-added food and nutraceutical applications (Figure 3). Recently, many studies have demonstrated that the nutrient and bioactive composition of hemp contributes to the prevention and treatment of several ailments suggesting its potential as a valuable functional food ingredient. This review sought to highlight these advances in understanding the medical, nutritional, and nutraceutical benefits of industrial hemp. The ease of production and suitability to many climatic and geographical locations are assets to the expansion of this industrial crop. Due to its versatility, breeding of hemp is underway in many universities and breeding centers across North America and Europe to develop high-yielding varieties for both fiber and oil seed production. This will help standardize varieties across different growing regions, thus maintaining quality and reducing disease and insect pressure. The controversial association of industrial hemp with medical Cannabis has also slowed expansion efforts. Therefore, breeding of hemp to clearly differentiate it from medical Cannabis may accelerate its development and consumer acceptance, as well as ease regulatory barriers of the crop.

A lot of advances have also been made in the extraction technologies of hemp seed oil and its nutraceutical benefits. However, there is still no industry consensus on the best methods of extraction, as it depends on the scale of production and end-use. The development of standardized processing guidelines for hemp seed and hemp seed oil will help ensure stringent quality control. There are opportunities in food innovation through the incorporation of hemp seed oil and its constituents, especially PUFA and CBD, in mainstream value-added and supplemented food products. Also, there is potential for the use of hemp processing byproducts in various food, feed, and industrial applications.

For innovation of novel hemp-derived food ingredients and nutraceuticals requires precise identification and quantification of major bioactives and standardization of the products. The analytical methods required for bioactives such as CBD need to be standardized. To ensure the authenticity and safety of hemp-derived food and nutraceuticals, it is important to quantify the amount of THC in the final product and includes it in the label. For example, in North America and most of Europe, to classify as industrial hemp, THC content should not exceed 0.3% on a dry weight basis. If the regulatory agencies could make a requirement for declaring THC content, that will help the food and nutraceutical industry to stay away from complicated regulatory issues around medical Cannabis. Since the impact of CBD is dose-dependent, an acceptable limit of CBD to be determined for inclusion in the labels of nutraceuticals and dietary supplements. The manufacturers should be aware that CBD content may change from batch-to-batch due to the variations of sources of materials, growing conditions, and manufacturing. Future investigations should also be aimed at quantification of trace cannabinoids other than THC and CBD and exploring their pharmacological effects. The pharmacokinetics of these bioactives, when incorporated in different food matrices, need to be understood. The inclusion of the content of omega-3 PUFA and omega-6 PUFA and their ratio in the label is useful for consumers to recognize the benefits of hemp oil and other value-added food products.

Most of the health benefits-associated research of industrial hemp has been conducted under pre-clinical conditions. However, due to the possibility of concentrating bioactive phytochemicals during the manufacturing process, the industry should pay attention to the dosing to optimize the potential health benefits and avoid possible safety concerns. There is a need to conduct appropriately designed, randomized, placebo-controlled, double-blind clinical studies on the effects of hemp-derived functional food ingredients and products, dietary supplements, and nutraceuticals on the promotion of human health. The hemp seed oil has potential as a nutraceutical due to the desired ratio of omega-6 PUFA to omega-3 PUFAs, and the bioactive CBD. Future research should focus on exploring other bioactive phytochemicals of industrial hemp, such as polyphenols and isoprenoids. The contribution of polyphenols and isoprenoids of hemp to the sensory quality, shelf life, and health benefits of the final products still to be understood. Overall, the hemp industry is starting to flourish across the globe. Regulatory agencies need to distinguish industrial hemp from medical Cannabis (marijuana), so the economic potential of industrial hemp as a sustainable source of value-added functional food ingredients and nutraceutical products can be realized.

## Figures and Tables

**Table 1:** Important major and minor constituents of hemp seed and hemp seed oil.

Product	Compound	Content	References
Hemp seed	Carbohydrate	20–30 ^†^; 27.6 ^†^	[3,35]
	Crude fat	25–35 ^†^; 33.2 ^†^; 30.4 ^†^; 31.1 ^†^	[2,35,36,37]
	Crude protein	20–25 ^†^; 24.8 ^†^; 24.9 ^†^; 24.0 ^†^; 27.3 ^†^	[2,3,35,36,37]
	Neutral detergent fiber	37.2 ^†^; 32.1 ^†^; 38.1 ^†^	[2,36,37]
	Acid detergent fiber	23.5 ^†^; 29.6 ^†^	[2,36]
	Ash	5.6 ^†^; 5.8 ^†^; 4.8 ^†^; 5.9 ^†^	[2,3,36,37]
Hemp seed oil	Cannabidiol (CBD)	10 ^‡^; 4.18–243.68 ^‡^	[43,44]
	Linoleic acid (omega-6 PUFA)	52–62 ^§^; 53.4 ^§^; 16.84 ^†^; 56.2 ^¶^; 56.07 ^§^	[2,41,43,44,45]
	Alpha-linolenic acid (omega-3 PUFA)	12–23 ^§^; 15.1 ^§^; 6.8 ^†^; 17.2 ^¶^; 15.98 ^§^	[2,41,43,44,45]
	Beta-tocopherol	6 ^‡^; 1.6 ^‡^; 0.64 ^‡^	[41,45,46]
	Gamma-tocopherol	733 ^‡^; 216.8 ^‡^; 91.57 ^‡^	[41,45,46]
	Alpha-tocopherol	34 ^‡^; 18.2 ^‡^; 19.74 ^‡^	[41,45,46]
	Delta-tocopherol	25 ^‡^; 12.0 ^‡^; 2.09 ^‡^	[41,45,46]

^†^, % Hemp seed fresh weight; ^‡^, mg/kg Hemp seed oil; ^§^, % Total fatty acids; ^¶^, % Hemp seed oil. PUFA, polyunsaturated fatty acid.
